# Gut Microbiota and Probiotics in Perioperative Management: A Narrative Review

**DOI:** 10.7759/cureus.68404

**Published:** 2024-09-01

**Authors:** Madhuri Kurdi, Sukhminder J. S Bajwa, Ridhima Sharma, Ripon Choudhary

**Affiliations:** 1 Department of Anaesthesiology, Karnataka Medical College and Research Institute, Hubballi, IND; 2 Department of Anaesthesiology, Gian Sagar Medical College and Hospital, Patiala, IND; 3 Department of Anaesthesiology, All India Institute of Medical Sciences, Nagpur, IND; 4 Department of Anaesthesiology, Datta Meghe Medical College and Research Institute, Nagpur, IND

**Keywords:** synbiotics, probiotics, postoperative, perioperative, pain, microbiota, delirium, cognitive dysfunction, brain-gut axis

## Abstract

The human gut is the abode of several complex and diverse microbes. It is a fact that the human brain is interconnected with the spinal cord and sense organs; however, there is also a possibility of a connection between the brain and the gut microbiome. The human gut can be altered in various ways, the principal method being the intake of prebiotics, probiotics and synbiotics. Can this alteration in the gut microbiome be clinically utilised in the perioperative period? We conducted a literature search related to this topic using databases and search engines (Medical Literature Analysis and Retrieval System Online {MEDLINE}, Embase, Scopus, PubMed and Google Scholar). The search revealed some preclinical and clinical studies in animals and humans that demonstrate the alteration of the gut microbiome with the use of anxiolysis, probiotics/prebiotics and other perioperative factors including opioids, anaesthetics and perioperative stress. The significant effects of this alteration have been seen on preoperative anxiety and postoperative delirium/cognitive dysfunction/pain. These effects are described in this narrative review, which opens up newer vistas for high-quality research related to the gut microbiome, gut-brain axis, the related signaling pathways and their clinical application in the perioperative period.

## Introduction and background

The gut of living beings has always served as a cosy home to microorganisms. These microorganisms are of traditional clinical interest as those that need to be replaced during an antibiotic course for diarrhoea and those that are responsible for sepsis due to translocation through a damaged intestinal mucosal barrier. Nevertheless, probiotics and prebiotics can alter the microbial flora of the gut, and certain strains of probiotics have been shown to confer health benefits [1]. Further, in the last few years, microbes in the gut have received special attention from researchers [2-7]. Do they have a connection with the brain? Which part of the host brain are they connected, and how can this be utilised for anaesthesia, pain and perioperative management? Can we manipulate the microbe families residing in the human gut to derive therapeutic perioperative benefits? In order to address all these questions, we conducted a literature search using databases and search engines (Medical Literature Analysis and Retrieval System Online {MEDLINE}, Embase, Scopus, PubMed and Google Scholar) from 30th January 2024 to 31st March 2024. All four authors were involved in the literature search.

We used key terms such as gut microbiota, probiotics, prebiotics, perioperative care, anaesthesia, surgery, postoperative care, preoperative care, anxiety, stress and pain management. We manually searched the articles for cross-references of interest. Amongst the articles (narrative and systematic review articles, original research articles and letters to the editor), the information that was interesting and that would help fill the gaps in the anaesthesiologist’s knowledge was selected. We conducted a narrative review than a systematic review because our research question is broad and exploratory and there is a lack of high-quality studies available on the topic.

## Review

Gut microbiota

The gut microbiota consists of a complex and diverse collection (approximately 1014) of organisms such as bacteria, archaea, yeast, single-celled eukaryotes and helminth parasites or viruses or both [1,8]. The bacteria are about 100 trillion in number and include more than 100 prevalent species and 160 species per individual [9]. The gut microbiome is a microecological system in which *Bacteroides*, *Firmicutes* and *Acinetobacter* dominate, while *Proteobacteria* and *Verrucomicrobia* are found in lesser populations [10]. Soon after birth, the composition of gut microbiota begins to take shape. The microbiota varies in composition depending on the location along the gastrointestinal tract (GIT) (oesophageal, gastric, proximal intestinal or distal intestinal) and axial depth (mucosal versus luminal) [11]. The large bowel harbours the maximum number of organisms compared to the stomach, duodenum and jejunum [12]. The microbial composition is distinct for every individual and remains constant over time. However, the major phyla of organisms can vary in proportion. The alteration of the gut microbiome and the composition and proportion of the bacteria depends on non-modifiable factors such as genetics, ageing, gender, race and ethnicity [13] and modifiable factors such as environmental changes, diet (mainly carbohydrate or noncarbohydrate and the amount of micronutrients in the diet such as polyphenols), antibiotic use, travel, psychiatric/physical stress, radiation, altered gastrointestinal tract (GIT) peristalsis, GIT infection and surgery [14]. The mode of delivery (vaginal or caesarean section), breast/formula feeding, weaning, diet, antibiotic intake, infections and stress can influence the gut microbiome in early life [15,16]. The oral intake of probiotics, prebiotics and synbiotics can alter the gut microbiome or flora [13]. Probiotics are live microorganisms that have beneficial health effects in the host when consumed or applied in sufficient quantities. Prebiotics are food ingredients that act as food for the growth and activity of selective human microflora in the colon. They escape digestion in the upper gastrointestinal (GI) tract. Synbiotics are a combination of pre- and probiotics [17].

The microbes live in a significant homeostatic relationship within the host gut, and this homeostasis is regulated by the intestinal gut bacteria and immune system [18]. GI microbes can catabolise protein to amino acids and participate in the luminal conversion of amino acids to biogenic amines, immunomodulatory compounds, small signaling molecules, antimicrobial peptides or bacteriocins that confer resilience or resistance to infection by enteric pathogens [13]. Gut microbiota excretes butyrate and small proteins such as occludin, claudin and zonula occludens in tight junctions between epithelial cells in the gut and help maintain desmosomes. Gut microbiota induces reactive oxygen species (ROS) production by epithelial cells. The ROS promote the healing of epithelial cells of a damaged intestinal wall.

Microbiota-gut-brain axis

The microbiota-gut-brain axis includes multiple organs such as the brain, glands, gut, immune cells and intestinal microbiota, which bidirectionally communicate to maintain homeostasis [19]. The vagus nerve is said to be a key communication between the gut microbiomes and the central nervous system [2]. The gut microbiota can directly or indirectly activate the vagus nerve. Hormones, neurochemicals and neurotransmitters such as dopamine, 5-hydroxytryptamine (5-HT), acetylcholine, gamma-aminobutyric acid (GABA) and bioactive peptides synthesised by the gut microbiome and enteroendocrine cells enter the brain via the intestinal wall and circulatory system and act on various areas of the brain or directly act on the ascending pathway of the vagus nerve to help in the regulation of transmission of brain signals [3]; e.g. dopamine produced by *Bacillus* and *E. coli* acts on the midbrain cortex pathway to participate in cognitive-related neuronal signal transduction through dopamine receptors [4]; 5-HT produced by *E. coli* and enterochromaffin cells (ECCs) can regulate pain and cognition [5].

Short-chain fatty acids (SCFAs) such as butyrate, acetate, lactate and propionate, which are produced by intestinal microbes through the fermentation of dietary fibre and carbohydrates, enter the circulatory system and can mediate interactions with the gut-brain signaling pathways [6]. SCFAs regulate 5-HT release from the ECCs [7]. Bacteria such as *Prevotella*, *Fusobacterium*, *Enterococcus casseliflavus*, *Escherichia* and *Bacteroides* produce tryptophan, which crosses the blood-brain barrier and allows serotoninergic neurotransmission in the brain [20].

Factors in the perioperative period that can affect the gut microbiome

Both intestinal and non-intestinal surgery can cause changes in the gut microbiota [21]. Antibiotics are most commonly used in the perioperative period, and their long-term use can lead to disturbances in the gut microbiome by reducing bacterial diversity and enhancing the colonisation of pathogenic and resistant bacteria [22]. Preoperative mechanical bowel cleansing includes the use of cleansing preparations that osmotically increase the volume of matter within the gut, washing out luminal contents including gut bacteria. Also, the increase in bowel movements flushes out bacteria incapable of adhering to the gut mucosa [23]. A study reported a decrease in the members of *Clostridium* cluster IV and increases in the members of *Clostridium* cluster XIVa and *Proteobacteria*, *Fusobacterium* and bacteria related to *Dorea formicigenerans*. This decrease lasted up to 14-28 days [24,25].

Preoperative fasting and the lack of enteral nutrition postoperatively can also affect gut microbiota [26]. The stress of surgery and the disruption of tissue homeostasis can lead to changes in the gut microbiome. The exposure of some species of gut microbiota to oxygen can lead to a decrease in good obligate anaerobes such as *Bacteroides* and an increase in ‘bad’ facultative anaerobes such as *Enterococcus* [27]. Tissue ischaemia due to the permanent or temporary occlusion of gut vessels can lead to a decrease in gut microbiota. There is progressive recovery of the microbes on reperfusion as observed in rats [28].

The length of surgical line incision, the type of suture used [29] and the degree of abdominal wall trauma may alter the degree of inflammation and the resultant catabolism and the production of catabolic hormones such as catecholamines and cortisol. Noradrenaline is known to interact with *E. coli* and *Pseudomonas aeruginosa* quorum-sensing receptors and activate the transcription of some microbial genes of virulent phenotypes [30].

Perioperative pharmacological agents such as opioids, antibiotics, acid-suppressing agents and the lack of gut motility can produce gut microbial dysbiosis [31].

Experimental models have demonstrated a significant alteration in the gut microbiome after exposure to anaesthetics, even after short-term exposure. For example, the levels of genera *Bacteroides*, *Alloprevotella* and *Akkermansia* have been found to be elevated, and the levels of *Lactobacillus* were reduced up to two weeks following sevoflurane anaesthesia in mice [32]. Similarly, intravenous propofol for three hours in rats has been found to produce reduction in *Prevotella*, *Alloprevotella* and *Lactobacillus* up to two weeks [33]. Isoflurane 1.4% of two-hour duration has been found to produce alterations in gut microbe composition in mice [34]. Morphine (subcutaneous pellets of 30 mg) administered in mice was found to result in significant alterations in the gut microbiome on the first day after administration itself [35]. There is a bidirectional interaction amongst opioids, opioid regulators and the microbiome. Opioids have been found to modulate the diversity of the gut microbiome, which in turn affects the host response to opioids [35]. Zhou et al. found that multiple exposures of neonatal Sprague Dawley rats to sevoflurane and surgery lead to subsequent anxiety-like behaviour in later life and changes in gut microbiota and stress hormone levels [36]. The gut microbiome can affect sensitivity to anaesthetics in animals; e.g. germ-free mice when exposed to phenobarbital displayed much faster recovery than similarly exposed specific pathogen-free mice [37].

A study in a mouse model demonstrated that spinal anaesthesia has protective effects against dextran sodium sulfate-induced colitis by increasing the number of Bacteroidetes in the colon and reducing the clinical symptoms of the mice such as the loss of body weight. It produced an improvement in inflammatory response and in the intestinal barrier functions [38].

The role of gut microbiota and probiotics in relieving stress and anxiety

Studies have shown that gut microbiota plays an influential role in many stress-related conditions [39,40]. A 12-week randomised, double-blind and placebo-controlled human trial demonstrated the clinical efficacy of the probiotic *L. plantarum* P-8 in reducing stress/anxiety [41]. This was associated with the decreased plasma levels of interferon (IFN), tumour necrosis factor (TNF)-alpha and cortisol. A study to investigate the functional role of gut metagenomes in the decrease of anxiety and stress showed that 12-week probiotic supplementation enhanced the diversity of neurotransmitter synthesising/consuming species-level genome and the levels of some predicted microbial neuroactive metabolites such as SCFAs, GABA, arachidonic acid and sphingomyelin [42].

Probiotic intake twice a day two weeks before surgery in patients with laryngeal cancer was found to have relieved the degree of anxiety of the patients and the biochemical features of stress [43].

In a prospective double-blind randomised clinical trial in adult patients undergoing elective major surgery under regional anaesthesia or general anaesthesia, the study group patients received a probiotic formula containing *Lactobacillus* and *Bifidobacterium*, and the placebo group patients received a multivitamin capsule twice daily after lunch and dinner for four days before the scheduled surgery. The researchers found a significant reduction in perceived stress scale scores, salivary amylase levels and psychomotor vigilance task scores following probiotic treatment, thus showing that gut microbiome alteration with probiotics results in the lowering of psychiatric stress and sleep improvement in the preoperative surgical patient [7].

Gut microbes, probiotics and their effect on postoperative cognitive dysfunction (POCD) (neurocognitive disorders)

Gut microbiota can affect cognitive functions through their metabolites, the regulation of immunity, the vagus nerve and the enteroendocrine system [44]. Research has found that dopamine produced by *Bacillus* and *E. coli* participates in cognitive-related neuronal signal transduction through dopamine receptors acting on the midbrain cortex pathway [4]. *Escherichia coli* produces 5-HT, which can regulate cognitive processes [4]. When the gram-negative intestinal bacteria increase in number, lipopolysaccharide can induce the release of proinflammatory factors and produce memory deficits [45]. *Campylobacter* in mice models increased anxiety-like behaviour, while *Bifidobacterium* and *Lactobacillus* could reduce anxiety and depression [46].

It is said that neuroinflammation can lead to the development of cognitive dysfunction. Anaesthesia can alter the gut milieu, trigger neuroinflammation and thereby produce cognitive dysfunction [47,48]. It is suggested that probiotics have anti-inflammatory capabilities, and by decreasing the levels of systemic proinflammatory cytokines such as interleukin (IL)-1B, TNF-alpha, IL-6, IL-10 and IFN-gamma, postoperative cognitive dysfunction (POCD) can be reduced [49-51]. Studies in humans including a randomised controlled trial in elderly patients undergoing noncardiac surgery have found that the perioperative oral supplementation of probiotics (*Bifidobacterium longum*, *L. acidophilus *and *Enterococcus faecalis*) from hospital admission to discharge significantly reduced the incidence of POCD. The researchers attributed this to the limitation of peripheral inflammation and the stress response [52].

Gut microbes, probiotics and their effect on postoperative delirium (POD)

It is evident from some studies in animals and human beings that a close relationship might exist between the gut microbiota and postoperative delirium (POD). In a study, when mice undergoing abdominal surgery and anaesthesia were separated into non-POD and POD phenotypes postoperatively, major differences were observed in the composition of the gut microbiome of the two groups, at multiple taxonomic levels. POD mice showed significantly decreased levels of *Ruminiclostridium*,* Ruminococcaceae* UCG014 and *Desulfovibrio* compared to non-POD mice [53].

In an experimental study to determine the potential age-dependent changes in behaviours, it was found that anaesthesia (1.4% isoflurane for two hours)/surgery induced greater POD-like behaviour in 18-month-old than in nine-month-old mice. *Lactobacillus* and probiotic treatment could reduce these behavioural changes [21]. Zhang et al. reported a different gut microbiota composition in eight-week-old mice with POD-like behaviour compared to those without POD [53].

In a clinical study on elderly patients aged 65 years or older who had a knee/hip replacement or laminectomy under general/spinal anaesthesia, the observations suggested an association between *Parabacteroides distasonis* in the gut and POD [54].

Gut microbes and postoperative pain

Postoperative pain can be due to the pain of the surgical trauma that produces inflammation and visceral pain, e.g. due to gut distension or neuropathic pain [18]. Aberrant gut microbial profiles can promote inflammation. Probiotics can modulate the gut microbiota to reduce or prevent inflammatory pain by several mechanisms such as controlling proinflammatory signaling via the increased expression of anti-inflammatory cytokines, directly limiting the bioavailability of specific proinflammatory cytokines [55,56], e.g. IL-10, transforming growth factor beta 1 (TGF-β1) and TNF, affecting inflammatory cytokine levels by acting on the nuclear factor kappa B (NF-κB) and mitogen-activated protein kinase (MAPK) signaling pathways [57], and the production of neurotransmitters, e.g. GABA, which binds to its receptors on the dorsal root ganglion neurons, resulting in their depolarisation and thereby the inhibition of peripheral nociceptive transmission [58]. The production or maintenance of secretory immunoglobulin A induced by the use of pro-/synbiotics can also help in reducing local postoperative inflammation [59]. Probiotics have been found in studies on Wistar rats and BALB/c mice to promote early wound healing by speeding up angiogenesis, fibroblast proliferation and re-epithelialisation, and this could help in the early reduction of postoperative pain [60,61].

Visceral pain results from the activation of nociceptors of thoracic, pelvic or abdominal organs. Splanchnic organs are sensitive to distension, ischaemia and inflammation. Probiotics can modulate visceral pain by mechanisms such as stimulating the expression of receptors on epithelial cells that locally control the transmission of nociceptive stimuli to the gut neurons [62], the induction of a sustained increase in mu opioid receptor (OPRMI) mRNA expression in human HT-29 epithelial cells and a decrease in cannabinoid receptor 2 (CNR 2) mRNA expression, the regulation of local IL-13/T-helper 17 (Th17) immune activation [63] and pain modulation by the alteration of the levels of neurotransmitters serotonin, noradrenaline, dopamine and biogenic amines in the frontal cortex, subcortex, brain stem and cerebellum [64,65]. A meta-analysis has shown that the administration of pro-/synbiotics perioperatively decreases the incidence of abdominal distension, shortens the time to first flatus and improves intestinal motility in patients after colorectal surgery [66].

Gut microbiota has been found to regulate several metabolic and neuronal signaling pathways between the gut and the central nervous system that are associated with neuropathic pain. In a rat model of spared nerve injury, the transplantation of faecal microbiota from spared-nerve-injury rats to pseudo-germ-free mice altered the sensibility of neuropathic pain [67].

In a nested case-control study of 132 females undergoing surgery for breast cancer, the baseline gut microbiome comparison was significantly different amongst the 66 females who developed chronic post-surgical pain (CPSP) at three months, compared to those patients who were pain-free [68]. It has been suggested that the interaction between host peroxisome proliferator-activated receptors and the gut microbiota may modulate inflammatory and neuropathic pain [69].

Gut bacteria, probiotics and postoperative infective complications

Several studies and meta-analyses have shown that patients receiving probiotics and synbiotics perioperatively have lesser chances of developing infectious complications postoperatively including respiratory infections, urinary tract infections, surgical site infections and sepsis and are associated with a reduced postoperative hospital stay and a reduced duration of antibiotic therapy [17,70,71]. The reasons for this could be the improvement in immune response, improved wound healing due to improvement in vascularisation, epithelialisation, decreased microbial load and the inhibition of biofilm formation. It is also possible that this could be due to the release of the wound-healing hormone oxytocin, which is thought to be the main gut-brain-skin mediator and which is increased following probiotic supplementation [72]. The decrease in systemic sepsis could be due to the probiotic effect of maintaining tight intestinal junctions as shown by the increased expression of tight junction proteins such as claudin-1, junctional adhesion molecule-1 and occludin in the epithelial cells of the intestine and the decreased ratio of lactulose to mannitol (L/M). Nevertheless, studies have shown that the intake of probiotics once daily six days before and 10 days after colectomy for colon cancer produced a reduced L/M ratio on the day before surgery and 10 days postoperatively [73,74].

Probiotics: availability, strains to be used and the route of administration

Probiotics are available in different combinations and brands. The most frequently used generation of probiotics are *Lactobacillus* and *Bifidobacterium* [75]. Other commonly used probiotic preparations are *Enterococcus*, *Streptococcus* and the yeast *Saccharomyces* [7]. The availability of probiotics varies from country to country, and there can be a lack of consistency between manufacturers and batches with reference to the density of bacteria, adhesion characteristics, stability and viability [76].

Whether to use a single strain or multiple strains of organisms is another dilemma. Multiple strains offer increased benefits since each strain offers its own benefits [77]. However, there are studies where a single strain has been used and has yielded good results. The best option would be to choose a multi-strain formula after careful consideration of strains and the intended benefit [77].

Whether to give the prebiotic preoperatively/postoperatively or both and for how many days are currently another dilemma. The duration of treatment is variable and shows large variations ranging from 15 days preoperatively to 30 days postoperatively with the median being 16 days. Nonetheless, the probiotic was administered for four days preoperatively in the Kurdi et al. study for preoperative anxiolysis [7]. Furthermore, the change in faecal microbial flora with the intake of prebiotics/synbiotics needs to be assessed in the patients, but it is difficult to completely analyse the faecal microbiome since there are so many bacteria. However, probiotics and prebiotics work in unison. They produce better health effects when consumed together as per logic and some authors [78].

The probiotics can be given orally for systemic effects or applied topically for wound healing. The typical dosages of probiotics vary based on the product. The common dosages for *Lactobacillus* and *Bifidobacterium* range from 10 to 20 billion colony-forming units orally per day for adults.

The quality and safety of probiotics

Probiotic foods are routinely consumed in large quantities as a part of the daily diet, e.g. yoghurt, kefir, sauerkraut, tempeh, kimchi, miso, kombucha, fermented pickles, traditional buttermilk (grandma’s probiotic: leftover liquid from making butter), natto and semihard cheeses. Probiotic formulations are also available on the counter in pharmacy shops and online, without a doctor’s prescription. This means that they are relatively safe. Several systematic reviews on probiotics have concluded that probiotics are overall safe [79,80]. Most researchers working on perioperative probiotics have not reported any side effects [7]. There are some reported cases of *Lactobacillus* bacteraemia [81,82]. However, *Lactobacillus* bacteraemia has an infrequent prevalence and is associated with a higher risk of mortality and risk factors, including severe underlying diseases, immune system suppression, admission to intensive care units, the presence of prosthetic cardiac valves, short gut syndrome, organ transplant and the use of central venous catheters [83]. *Lacticaseibacillus rhamnosus *GG, *Lactiplantibacillus plantarum* and *Lacticaseibacillus paracasei* have been directly linked with bacteraemia [84].

There is a possibility that in case of a break in the cold chain/a person with a high degree of acidity in the stomach or slow digestion, the microbes in the oral probiotic formulas might be killed before they reach the large intestines alive. Abdominal bloating, constipation and thirst are some reported rare side effects of probiotics [7].

The monitoring of probiotic treatment

The probiotic species can be tested in the gastric aspirates after a median of 13 days of treatment, in oropharyngeal and tracheal cultures and in stool cultures [12]. Although easily acquired, stool specimens are considered to be poorly representative of the upper GI tract and the mucosa-associated microbiota [13].

Limitations of current studies on probiotics

Though studies and meta-analyses related to gut bacteria and the perioperative clinical use of probiotics are being conducted and published, many of the studies have small sample sizes. Many of the studies are experimental studies in rats and mice.

The variation in microbiomes, study population, surgeries and probiotics pose difficulties in comparison. The general health and basal gut microbiome of every patient scheduled for surgery will not be the same.

Heterogeneity has been reported amongst the different probiotic formulas used with their various combinations, the dose (the amount of bacteria) administered, the duration of treatment, the route of administration, the timing of administration (peri-/pre-/postoperative) and the protocol applied for administration [85,86]. This diversity poses limitations on the strength of the conclusions drawn and the ability to pool results in a meta-analysis.

Though the range of probiotic species is increasing for research purposes and the number of indications is also increasing, the rationale behind the use of particular probiotic strain/strains is not clear. Further, this review has some limitations. The selection criteria for the articles discussed in this review, though stated, are non-explicit. There is potential for a selection bias. Nevertheless, this is possible in a narrative review.

Areas of future research

The components of the probiotic-gut-microbiome-brain axis and the interaction between various components of the axis need to be examined and understood more in-depth. The superiority of one probiotic species over the other, the optimal dosage, the duration, the timing and the route of administration need to be further explored.

The mechanism of action of the probiotics and gut bacteria in the molecular mechanisms underlying the modulation of POCD, POD, pain and anxiety may not be as simple as depicted. Well-designed, adequately powered randomised controlled trials on the perioperative applications of pro-/prebiotics and gut microbes are needed. The possibility of the specific signature of the gut microbiota as biomarkers of pain, anxiety and POCD needs to be further researched. More preclinical and clinical studies need to be conducted to investigate the role of gut bacteria and prebiotics in perioperative anxiolysis, pain modulation and the prevention of postoperative emergence, POCD and POD. Faecal microbial assays can enable one to understand the gut microbial flora of a person, and this can be used as a preoperative test in the coming days to estimate the risk of anxiety, stress, POCD and pain in the individual and thereby to plan the appropriate anaesthesia regimen including the choice of drugs and their dosages as in precision medicine. A faecal microbial transplant can be done preoperatively to alter the gut microbiome to favour perioperative outcomes. Studies comparing probiotics to synbiotics for perioperative application can be conducted.

## Conclusions

The evidence gathered in this review suggests that the gut microbiome may be altered with the use of anxiolytics, probiotics/prebiotics and other perioperative factors including opioids, anaesthetics and perioperative stress. Significant effects of this alteration may be seen on preoperative anxiety, POD, POCD and postoperative pain.

Nevertheless, further high-quality intensive research related to the gut-brain axis and the clinical application of gut microbes in the perioperative period is needed.

## References

[1] Karczewski J, Troost FJ, Konings I, Dekker J, Kleerebezem M, Brummer RJ, Wells JM (2010). Regulation of human epithelial tight junction proteins by Lactobacillus plantarum in vivo and protective effects on the epithelial barrier. Am J Physiol Gastrointest Liver Physiol.

[2] Diaz Heijtz R, Wang S, Anuar F (2011). Normal gut microbiota modulates brain development and behavior. Proc Natl Acad Sci U S A.

[3] Mittal R, Debs LH, Patel AP (2017). Neurotransmitters: the critical modulators regulating gut-brain axis. J Cell Physiol.

[4] Hsieh TH, Kuo CW, Hsieh KH (2020). Probiotics alleviate the progressive deterioration of motor functions in a mouse model of Parkinson’s disease. Brain Sci.

[5] Yano JM, Yu K, Donaldson GP (2015). Indigenous bacteria from the gut microbiota regulate host serotonin biosynthesis. Cell.

[6] Dalile B, Van Oudenhove L, Vervliet B, Verbeke K (2019). The role of short-chain fatty acids in microbiota-gut-brain communication. Nat Rev Gastroenterol Hepatol.

[7] Kurdi MS, Ramaswamy AH, Kumar LA, Choukimath SM, Jangi AA (2021). Use of a non-invasive biomarker salivary alpha-amylase to assess the role of probiotics in sleep regulation and stress attenuation in surgical patients: a randomised double-blind clinical trial. Indian J Anaesth.

[8] Sommer F, Bäckhed F (2013). The gut microbiota--masters of host development and physiology. Nat Rev Microbiol.

[9] Qin J, Li R, Raes J (2010). A human gut microbial gene catalogue established by metagenomic sequencing. Nature.

[10] Tojo R, Suárez A, Clemente MG, de los Reyes-Gavilán CG, Margolles A, Gueimonde M, Ruas-Madiedo P (2014). Intestinal microbiota in health and disease: role of bifidobacteria in gut homeostasis. World J Gastroenterol.

[11] Eckburg PB, Bik EM, Bernstein CN (2005). Diversity of the human intestinal microbial flora. Science.

[12] Crooks NH, Snaith C, Webster D, Gao F, Hawkey P (2012). Clinical review: probiotics in critical care. Crit Care.

[13] Hollister EB, Gao C, Versalovic J (2014). Compositional and functional features of the gastrointestinal microbiome and their effects on human health. Gastroenterology.

[14] Hawrelak JA, Myers SP (2004). The causes of intestinal dysbiosis: a review. Altern Med Rev.

[15] Vuong HE, Yano JM, Fung TC, Hsiao EY (2017). The microbiome and host behavior. Annu Rev Neurosci.

[16] Schmidt TS, Raes J, Bork P (2018). The human gut microbiome: from association to modulation. Cell.

[17] Chowdhury AH, Adiamah A, Kushairi A (2020). Perioperative probiotics or synbiotics in adults undergoing elective abdominal surgery: a systematic review and meta-analysis of randomized controlled trials. Ann Surg.

[18] Fyntanidou B, Amaniti A, Soulioti E (2023). Probiotics in postoperative pain management. J Pers Med.

[19] Keshavarzian A, Green SJ, Engen PA (2015). Colonic bacterial composition in Parkinson's disease. Mov Disord.

[20] Ustianowska K, Ustianowski Ł, Machaj F (2022). The role of the human microbiome in the pathogenesis of pain. Int J Mol Sci.

[21] Liufu N, Liu L, Shen S (2020). Anesthesia and surgery induce age-dependent changes in behaviors and microbiota. Aging (Albany NY).

[22] Modi SR, Collins JJ, Relman DA (2014). Antibiotics and the gut microbiota. J Clin Invest.

[23] Jalanka J, Salonen A, Salojärvi J (2015). Effects of bowel cleansing on the intestinal microbiota. Gut.

[24] Drago L, Toscano M, De Grandi R, Casini V, Pace F (2016). Persisting changes of intestinal microbiota after bowel lavage and colonoscopy. Eur J Gastroenterol Hepatol.

[25] Drago L, Valentina C, Fabio P (2019). Gut microbiota, dysbiosis and colon lavage. Dig Liver Dis.

[26] Liang W, Yang Y, Wang H (2019). Gut microbiota shifts in patients with gastric cancer in perioperative period. Medicine (Baltimore).

[27] Shogan BD, Smith DP, Christley S, Gilbert JA, Zaborina O, Alverdy JC (2014). Intestinal anastomotic injury alters spatially defined microbiome composition and function. Microbiome.

[28] Wang F, Li Q, Wang C, Tang C, Li J (2012). Dynamic alteration of the colonic microbiota in intestinal ischemia-reperfusion injury. PLoS One.

[29] Ioannidis A, Arvanitidis K, Filidou E (2018). The length of surgical skin incision in postoperative inflammatory reaction. JSLS.

[30] Kendall MM, Sperandio V (2016). What a dinner party! Mechanisms and functions of interkingdom signaling in host-pathogen associations. mBio.

[31] Kotzampassi K (2022). What surgeon should know about probiotics. Nutrients.

[32] Han C, Zhang Z, Guo N (2021). Effects of sevoflurane inhalation anesthesia on the intestinal microbiome in mice. Front Cell Infect Microbiol.

[33] Guo N, Zhang Z, Han C (2021). Effects of continuous intravenous infusion of propofol on intestinal flora in rats. Biomed Pharmacother.

[34] Yang S, Gu C, Mandeville ET (2017). Anesthesia and surgery impair blood-brain barrier and cognitive function in mice. Front Immunol.

[35] Wang F, Meng J, Zhang L, Johnson T, Chen C, Roy S (2018). Morphine induces changes in the gut microbiome and metabolome in a morphine dependence model. Sci Rep.

[36] Zhou X, Xu X, Lu D (2023). Repeated early-life exposure to anaesthesia and surgery causes subsequent anxiety-like behaviour and gut microbiota dysbiosis in juvenile rats. Br J Anaesth.

[37] Vuong HE, Hsiao EY (2017). Emerging roles for the gut microbiome in autism spectrum disorder. Biol Psychiatry.

[38] Hong Y, Zhao J, Chen YR, Huang ZH, Hou LD, Shen B, Xin Y (2022). Spinal anesthesia alleviates dextran sodium sulfate-induced colitis by modulating the gut microbiota. World J Gastroenterol.

[39] Foster JA, Rinaman L, Cryan JF (2017). Stress & the gut-brain axis: regulation by the microbiome. Neurobiol Stress.

[40] Rea K, Dinan TG, Cryan JF (2020). Gut microbiota: a perspective for psychiatrists. Neuropsychobiology.

[41] Lew LC, Hor YY, Yusoff NA (2019). Probiotic Lactobacillus plantarum P8 alleviated stress and anxiety while enhancing memory and cognition in stressed adults: a randomised, double-blind, placebo-controlled study. Clin Nutr.

[42] Ma T, Jin H, Kwok LY, Sun Z, Liong MT, Zhang H (2021). Probiotic consumption relieved human stress and anxiety symptoms possibly via modulating the neuroactive potential of the gut microbiota. Neurobiol Stress.

[43] Yang H, Zhao X, Tang S (2016). Probiotics reduce psychological stress in patients before laryngeal cancer surgery. Asia Pac J Clin Oncol.

[44] Alam A, Hana Z, Jin Z, Suen KC, Ma D (2018). Surgery, neuroinflammation and cognitive impairment. EBioMedicine.

[45] Jang SE, Lim SM, Jeong JJ, Jang HM, Lee HJ, Han MJ, Kim DH (2018). Gastrointestinal inflammation by gut microbiota disturbance induces memory impairment in mice. Mucosal Immunol.

[46] Goehler LE, Park SM, Opitz N, Lyte M, Gaykema RP (2008). Campylobacter jejuni infection increases anxiety-like behavior in the holeboard: possible anatomical substrates for viscerosensory modulation of exploratory behavior. Brain Behav Immun.

[47] Wen J, Ding Y, Wang L, Xiao Y (2020). Gut microbiome improves postoperative cognitive function by decreasing permeability of the blood-brain barrier in aged mice. Brain Res Bull.

[48] Han D, Li Z, Liu T (2020). Prebiotics regulation of intestinal microbiota attenuates cognitive dysfunction induced by surgery stimulation in APP/PS1 mice. Aging Dis.

[49] Schachter J, Martel J, Lin CS (2018). Effects of obesity on depression: a role for inflammation and the gut microbiota. Brain Behav Immun.

[50] Choi SH, Oh JW, Ryu JS, Kim HM, Im SH, Kim KP, Kim MK (2020). IRT5 probiotics changes immune modulatory protein expression in the Extraorbital lacrimal glands of an autoimmune dry eye mouse model. Invest Ophthalmol Vis Sci.

[51] Jiang XL, Gu XY, Zhou XX (2019). Intestinal dysbacteriosis mediates the reference memory deficit induced by anaesthesia/surgery in aged mice. Brain Behav Immun.

[52] Wang P, Yin X, Chen G (2021). Perioperative probiotic treatment decreased the incidence of postoperative cognitive impairment in elderly patients following non-cardiac surgery: a randomised double-blind and placebo-controlled trial. Clin Nutr.

[53] Zhang J, Bi JJ, Guo GJ (2019). Abnormal composition of gut microbiota contributes to delirium-like behaviors after abdominal surgery in mice. CNS Neurosci Ther.

[54] Zhang Y, Baldyga K, Dong Y (2023). The association between gut microbiota and postoperative delirium in patients. Transl Psychiatry.

[55] Menni A, Moysidis M, Tzikos G (2023). Looking for the ideal probiotic healing regime. Nutrients.

[56] Vanderwall AG, Milligan ED (2019). Cytokines in pain: harnessing endogenous anti-inflammatory signaling for improved pain management. Front Immunol.

[57] Thomas CM, Versalovic J (2010). Probiotics-host communication: modulation of signaling pathways in the intestine. Gut Microbes.

[58] Du X, Hao H, Yang Y (2017). Local GABAergic signaling within sensory ganglia controls peripheral nociceptive transmission. J Clin Invest.

[59] Li Y, Jin L, Chen T (2020). The effects of secretory IgA in the mucosal immune system. Biomed Res Int.

[60] Mohtashami M, Mohamadi M, Azimi-Nezhad M, Saeidi J, Nia FF, Ghasemi A (2021). Lactobacillus bulgaricus and Lactobacillus plantarum improve diabetic wound healing through modulating inflammatory factors. Biotechnol Appl Biochem.

[61] Dubey AK, Podia M, Priyanka Priyanka, Raut S, Singh S, Pinnaka AK, Khatri N (2021). Insight into the beneficial role of Lactiplantibacillus plantarum supernatant against bacterial infections, oxidative stress, and wound healing in A549 cells and BALB/c mice. Front Pharmacol.

[62] Rousseaux C, Thuru X, Gelot A (2007). Lactobacillus acidophilus modulates intestinal pain and induces opioid and cannabinoid receptors. Nat Med.

[63] Darbaky Y, Evrard B, Patrier S (2017). Oral probiotic treatment of Lactobacillus rhamnosus Lcr35(®) prevents visceral hypersensitivity to a colonic inflammation and an acute psychological stress. J Appl Microbiol.

[64] Kannampalli P, Pochiraju S, Chichlowski M (2014). Probiotic Lactobacillus rhamnosus GG (LGG) and prebiotic prevent neonatal inflammation-induced visceral hypersensitivity in adult rats. Neurogastroenterol Motil.

[65] Roman P, Abalo R, Marco EM, Cardona D (2018). Probiotics in digestive, emotional, and pain-related disorders. Behav Pharmacol.

[66] Tang G, Huang W, Tao J, Wei Z (2022). Prophylactic effects of probiotics or synbiotics on postoperative ileus after gastrointestinal cancer surgery: a meta-analysis of randomized controlled trials. PLoS One.

[67] Yang C, Fang X, Zhan G (2019). Key role of gut microbiota in anhedonia-like phenotype in rodents with neuropathic pain. Transl Psychiatry.

[68] Yao ZW, Yang X, Zhao BC (2022). Predictive and preventive potential of preoperative gut microbiota in chronic postoperative pain in breast cancer survivors. Anesth Analg.

[69] Maeda T, Kishioka S (2009). PPAR and pain. Int Rev Neurobiol.

[70] Veziant J, Bonnet M, Occean BV, Dziri C, Pereira B, Slim K (2022). Probiotics/synbiotics to reduce infectious complications after colorectal surgery: a systematic review and meta-analysis of randomised controlled trials. Nutrients.

[71] Tang G, Zhang L, Tao J, Wei Z (2021). Effects of perioperative probiotics and synbiotics on pancreaticoduodenectomy patients: a meta-analysis of randomized controlled trials. Front Nutr.

[72] Agnes A, Puccioni C, D'Ugo D, Gasbarrini A, Biondi A, Persiani R (2021). The gut microbiota and colorectal surgery outcomes: facts or hype? A narrative review. BMC Surg.

[73] Liu Z, Qin H, Yang Z (2011). Randomised clinical trial: the effects of perioperative probiotic treatment on barrier function and post-operative infectious complications in colorectal cancer surgery - a double-blind study. Aliment Pharmacol Ther.

[74] Liu ZH, Huang MJ, Zhang XW (2013). The effects of perioperative probiotic treatment on serum zonulin concentration and subsequent postoperative infectious complications after colorectal cancer surgery: a double-center and double-blind randomized clinical trial. Am J Clin Nutr.

[75] Isolauri E, Arvola T, Sütas Y, Moilanen E, Salminen S (2000). Probiotics in the management of atopic eczema. Clin Exp Allergy.

[76] Tuomola E, Crittenden R, Playne M, Isolauri E, Salminen S (2001). Quality assurance criteria for probiotic bacteria. Am J Clin Nutr.

[77] Bengmark S (2012). Pro- and synbiotics to prevent sepsis in major surgery and severe emergencies. Nutrients.

[78] Roberfroid M, Gibson GR, Hoyles L (2010). Prebiotic effects: metabolic and health benefits. Br J Nutr.

[79] Yang B, Wei J, Ju P, Chen J (2019). Effects of regulating intestinal microbiota on anxiety symptoms: a systematic review. Gen Psychiatr.

[80] Islam SU (2016). Clinical uses of probiotics. Medicine (Baltimore).

[81] Salminen MK, Tynkkynen S, Rautelin H (2002). Lactobacillus bacteremia during a rapid increase in probiotic use of Lactobacillus rhamnosus GG in Finland. Clin Infect Dis.

[82] Land MH, Rouster-Stevens K, Woods CR, Cannon ML, Cnota J, Shetty AK (2005). Lactobacillus sepsis associated with probiotic therapy. Pediatrics.

[83] Abgrall S, Joly V, Derkinderen P, Decré D, Carbon C, Yeni P (1997). Lactobacillus casei infection in an AIDS patient. Eur J Clin Microbiol Infect Dis.

[84] Kullar R, Goldstein EJ, Johnson S, McFarland LV (2023). Lactobacillus bacteremia and probiotics: a review. Microorganisms.

[85] Yang Z, Wu Q, Liu Y, Fan D (2017). Effect of perioperative probiotics and synbiotics on postoperative infections after gastrointestinal surgery: a systematic review with meta-analysis. JPEN J Parenter Enteral Nutr.

[86] Liu PC, Yan YK, Ma YJ (2017). Probiotics reduce postoperative infections in patients undergoing colorectal surgery: a systematic review and meta-analysis. Gastroenterol Res Pract.

